# Evolution of SARS-CoV-2 antibody repertoire after successive mRNA vaccinations under immunosuppressive treatment

**DOI:** 10.1016/j.ebiom.2025.105620

**Published:** 2025-02-25

**Authors:** Jim B.D. Keijser, Eileen W. Stalman, Luuk Wieske, Maurice Steenhuis, Koos P.J. van Dam, Laura Y.L. Kummer, Zoé L.E. van Kempen, Joep Killestein, Adriaan G. Volkers, Sander W. Tas, Laura Boekel, Gerrit J. Wolbink, Laura Fernandez Blanco, Niels J.M. Verstegen, Sofie Keijzer, Gerard van Mierlo, Olvi Cristianawati, Arend J. Boogaard, Karlijn van der Straten, Jacqueline van Rijswijk, Marit J. van Gils, Anja ten Brinke, S. Marieke van Ham, Taco W. Kuijpers, Filip Eftimov, Theo Rispens, T2B! Immunity against SARS-CoV-2 study group, Anneke J. Van der Kooi, Joop Raaphorst, Mark Löwenberg, R. Bart Takkenberg, Geert R.A.M. D'Haens, Phyllis I. Spuls, Marcel W. Bekkenk, Annelie H. Musters, Nicoline F. Post, Angela L. Bosma, Marc L. Hilhorst, Yosta Vegting, Frederike J. Bemelman, Alexandre E. Voskuyl, Bo Broens, Agner R. Parra Sanchez, Cécile A.C.M. Van Els, Jelle De Wit, Abraham Rutgers, Karina De Leeuw, Barbara Horváth, Jan J.G.M. Verschuuren, Annabel M. Ruiter, Lotte Van Ouwerkerk, Diane Van der Woude, Renée C.F. Van Allaart, Y.K. Onno Teng, Pieter Van Paassen, Matthias H. Busch, Papay B.P. Jallah, Esther Brusse, Pieter A. Van Doorn, Adája E. Baars, Dirk Jan Hijnen, Corine R.G. Schreurs, W. Ludo Van der Pol, H. Stephan Goedee, Koos A.H. Zwinderman, Rivka De Jongh, Carolien E. Van de Sandt, Lisan H. Kuijper, Mariël C. Duurland, Ruth R. Hagen, Jet Van den Dijssel, Christine Kreher, Amélie V. Bos, Virginia Palomares Cabeza, Veronique A.L. Konijn, George Elias, Elham S. Mirfazeli

**Affiliations:** a Department of Immunopathology, Sanquin Research and Landsteiner Laboratory, Amsterdam, the Netherlands; b Amsterdam Institute for Immunology and Infectious Diseases, Amsterdam, the Netherlands; c Department of Neurology and Neurophysiology, Amsterdam Neuroscience, Amsterdam UMC, University of Amsterdam, Amsterdam, the Netherlands; d Department of Clinical Neurophysiology, St Antonius Hospital, Nieuwegein, the Netherlands; e Department of Neurology, Amsterdam UMC, Amsterdam, the Netherlands; f Department of Gastroenterology and Hepatology, Amsterdam UMC, University of Amsterdam, Amsterdam, the Netherlands; g Amsterdam Rheumatology and Immunology Center, Department of Rheumatology and Clinical Immunology, Amsterdam UMC, University of Amsterdam, Amsterdam, the Netherlands; h Amsterdam Rheumatology and Immunology Center, Department of Rheumatology, Reade, Amsterdam, the Netherlands; i Department of Medical Microbiology and Infection Prevention, Amsterdam UMC, University of Amsterdam, Amsterdam, the Netherlands; j Swammerdam Institute for Life Sciences, University of Amsterdam, Amsterdam, the Netherlands; k Department of Pediatric Immunology, Rheumatology and Infectious Disease, Amsterdam UMC, University of Amsterdam, Amsterdam, the Netherlands; l Department of Molecular Cell Biology and Immunology, Amsterdam UMC location Vrije Universiteit Amsterdam, Amsterdam, The Netherlands

**Keywords:** Antibody repertoire, mRNA vaccines, Serology, SARS-CoV-2, Autoimmune disease

## Abstract

**Background:**

Repeated antigen exposure can result in a shifting antibody repertoire. The mechanisms by which this occurs and consequences for cross-variant protection against evolving pathogens remain incompletely understood, particularly in the context of immunosuppressive treatments used in patients with immune-mediated inflammatory diseases (IMID).

**Methods:**

To investigate this, we characterised longitudinal changes in the anti-SARS-CoV-2 antibody repertoire over the course of three SARS-CoV-2 mRNA vaccinations in patients with IMIDs treated with methotrexate (MTX) and/or tumour necrosis factor-inhibitors (TNFi), anti-CD20 monoclonal antibodies, no systemic therapy, and healthy controls (total N = 878). We determined serum antibody titres against the receptor-binding domain (RBD) of Wuhan-Hu-1 (WH1) and Omicron BA.1 spike proteins, and assessed ratios thereof between groups as a proxy for cross-reactivity.

**Findings:**

We observe emerging anti-BA.1 RBD reactivity over time, notably following a third vaccination. This may be partly explained by affinity maturation, as evaluated by inhibition of ACE2-RBD interactions. Similar trends were seen in patients treated with MTX and/or TNFi, but not in patients on anti-CD20 therapy. SARS-CoV-2 infection prior to vaccination accelerated these effects initially while leading to comparable results after three vaccinations.

**Interpretation:**

MTX and TNFi do not qualitatively alter the evolution of the antibody repertoire in response to repeated antigen exposure, whereas anti-CD20 does. These insights may help to optimise vaccination strategies for patients with immune-mediated inflammatory diseases.

**Funding:**

This study was supported by 10.13039/501100001826ZonMw (The Netherlands Organization for Health Research and Development) and SGF (Collaborating Health Funds).

**Research in context**

**Evidence before this study**

Repeated mRNA immunization with the spike protein of the ancestral Wuhan-Hu-1 (WH1) strain of SARS-CoV-2 improves humoural immunity against subsequent variants despite considerable mutation. Studies in healthy individuals and mice indicate this results from the combined effects of affinity maturation and a shift towards B cell clones that target epitopes in conserved regions of the spike protein. This shift is thought to involve competition for antigen binding between germinal centre B cells and existing serum antibodies from a sufficiently robust previous immune response, which may be compromised in immunosuppressed individuals. We previously investigated a number of commonly used immunosuppressive medications and their effects on short-term antibody responses against the immunodominant receptor-binding domain (RBD) of the WH1 spike protein, reporting moderately diminished titres for some and severe decreases or no seroconversion for others. How these drugs may affect long-term trends in cross-reactivity remains incompletely understood.

**Added value of this study**

We demonstrate in a large and disease-overarching prospective cohort that immune-mediated inflammatory disease patients treated with methotrexate (MTX) and/or tumour necrosis factor (TNF) inhibitors (TNFi) show similar trends in cross-reactivity to Omicron subvariant BA.1. However, such repertoire evolution does not occur in patients treated with anti-CD20 monoclonal antibodies.

**Implications of all the available evidence**

Patients treated with MTX and/or TNFi likely respond to vaccination in a qualitatively similar manner as healthy individuals, whereas patients treated with anti-CD20 do not, and booster doses may not elicit a protective degree of cross-reactivity while effective drug concentration is maintained. Our findings provide insights that may help to optimise vaccination strategies for patients with immune-mediated inflammatory diseases.

## Introduction

A hallmark of adaptive immunity is the exquisite specificity that allows a virtually endless variety of pathogens to be targeted. However, this specificity introduces the potential for immune evasion in the case of rapidly mutating pathogens. While the ideal antibody repertoire should therefore be reactive across possible variants, challenge by particular antigens may yield varying degrees of cross-reactivity depending on whether a response can be elicited against conserved regions of the antigen.

The SARS-CoV-2 pandemic has further exemplified this fact. While a historic research and development effort resulted in rapid deployment of numerous vaccines, with novel mRNA vaccines in particular providing >90% protection against infection by the ancestral Wuhan-Hu-1 (WH1) strain of SARS-CoV-2,^1^^,^^2^ numerous variants have since proven partially evasive to previous vaccine- or infection-induced antibodies. The Omicron (BA.1) variant in particular prompted booster mRNA vaccination campaigns around the world. Such repeat vaccinations have been protective against severe disease, and to a lesser degree against infection by Omicron-lineage variants, despite the significant degree of mutation from the mRNA vaccines’ WH1 spike Immunogen.^3^^,^^4^

Previous studies suggest this improved protection against newer variants is not merely a matter of restoring waned titres, but also stems from a change in the antibody repertoire that confers greater cross-reactivity. These changes have been reported to be driven both by ongoing maturation of clones already present after second vaccination, as well as newly expanded clones that show a preference towards epitopes less affected by BA.1 mutations.^5^, ^6^, ^7^ Thus, this evolution of the antibody repertoire depends on long-term B cell function.

We previously demonstrated that with regard to short-term anti-WH1 receptor-binding domain (RBD) responses, patients with immune-mediated inflammatory diseases (IMIDs) treated with a variety of immunomodulating therapies including methotrexate (MTX) and TNF inhibitors (TNFi) had similar seroconversion rates and moderately reduced titres compared to healthy donors and patients not on immunosuppression.^8^ However, several “poor-responder” therapies were also identified, including anti-CD20 monoclonal antibodies. How the antibody repertoire develops over a longer period of time under these different modes of immunosuppression remains incompletely understood despite the particular importance of robust protection to vulnerable patients.

To study the impact of immunosuppressive agents on the evolution of the antibody repertoire following mRNA vaccination with BNT162b2 or mRNA-1273, we here analysed IgG responses against the WH1 RBD and BA.1 RBD and determined ratios as a titre-independent measure of cross-reactivity. Serum samples from patients with IMIDs treated with different immunosuppressive medication (MTX and/or TNFi, anti-CD20, or none) and healthy donors were collected longitudinally to assess the separate effects of time and repeated antigen challenge.

## Methods

### Study participants

Here we report on changes in the antibody repertoire after repeated vaccinations, which was a predefined secondary objective of the prospective Target-2-B! Immunity against SARS-CoV-2 vaccination cohort study. Details and full study protocol have been described previously^8^; in short, participants were over the age of 18 and diagnosed with any of a prespecified set of IMIDs, with no history of oncological or haematological disorders, receiving maintained treatment before and during the study period. For this substudy, we selected patients treated with MTX or TNFi monotherapy or MTX + TNFi combination therapy, and anti-CD20 monotherapy, to represent previously identified moderate and poor-responder treatment groups respectively.^8^ Healthy donors with no history of immunological disorders were also included (healthy controls, HC) as well as patients with IMIDs who were not on systemic immunosuppressive medications (disease controls, DC). This treatment-based grouping is unchanged from the original cohort, which was recruited in a sex- and age-matched manner. Differences between groups in participants’ underlying diseases remains as a notable confounder that cannot be controlled for in this observational context, though we previously found that treatment effects explained most of the variation in vaccination responses.^8^

Selected participants received two homologous doses of BNT162b2 or mRNA-1273 between April–July 2021, and a third mRNA dose that was not always homologous. Serum samples were collected prior to vaccination (V1_pre_), 28 days after the first and second doses (V1_post_; V2_post_), and prior to and 28 days after the third dose (V3_pre_; V3_post_) by at-home fingerprick (all timepoints) or venipuncture at a participating hospital (V1_pre_ only). Not all participants had samples available at every timepoint, as participants did not always notify the research team of upcoming or received vaccinations in a timely manner for sampling. Missing samples including loss to follow-up after V2_post_ were previously found to be comparable across groups and are thus regarded as missing at random.^8^

Participants were considered antigen-experienced before vaccination if positive for total anti-RBD antibodies at V1_pre_, positive for total anti-nucleocapsid protein (NP) antibodies at V1_post_ or V2_post_, or a positive SARS-CoV-2 PCR was reported at any time before vaccination.

### Ethics

This study was approved by the medical ethical committee of the Amsterdam UMC (2020.194; trial registry NL74974.018.20 and EudraCT 2021-001102-30). All participants provided written informed consent.

### Serological assays

#### Total antibody ELISAs

For qualitative detection of anti-SARS-CoV-2 antibodies resulting from infections, we performed in-house developed total antibody ELISAs against WH1 RBD and nucleocapsid protein (NP), as described previously.^9^

#### Isotype-specific serology

For (semi-)quantitative measurement of anti-SARS-CoV-2 antibodies, we performed in-house developed IgG-specific ELISAs against WH1 RBD, WH1 full spike (S), and BA.1 RBD, as well as an IgM-specific version against WH1 RBD. WH1 RBD IgG, WH1 S IgG and WH1 RBD IgM assays were calibrated using pooled plasma from convalescent healthy donors obtained in May 2020 when WH1 was dominant, and 99% specificity cutoffs were determined at 4, 6 and 0.5 AU/mL respectively as described previously.^9^, ^10^, ^11^

For calibration of the BA.1 RBD IgG assay, another pooled plasma calibrator was obtained from convalescent healthy donors in February 2022 when BA.1 was dominant in the Netherlands. This calibrator was also set to 100 AU/mL based on similar levels of IgG detection compared to WH1 RBD calibrator titrated onto WH1 RBD coat within the same experiment. BA.1 RBD for coating was produced as previously described for WH1 RBD.^9^ A 99% specificity cutoff was determined at 4 AU/mL based on N = 352 pre-2019 samples. Besides these calibrator and coat changes, the BA.1 RBD assay was performed as the previously described WH1 RBD assay.

Titre ratios for a given sample were calculated as its BA.1 RBD titre divided by its WH1 RBD titre. A given ratio was dropped from analysis if both of its contributing titre values were below seroconversion cutoffs, to avoid skewing from meaningless noise/noise comparisons. In the participant groups and timepoints shown, >75% of ratio values derive from two titre values that were above seroconversion cutoffs. Titre values below 1 AU/mL were set to 1 AU/mL for calculations.

For validation purposes, we performed site-specific antigen immobilization versions of our IgG ELISAs. S and RBD protein was biotinylated via AviTags already present in the constructs, using a birA ligase kit (Sigma–Aldrich) per manufacturer instructions. 100 μL/well of 0.5 μg/mL biotinylated antigen was coated onto streptatividin-coated plates (Thermo Fisher Scientific) for 1 h at room temperature while shaking. The assay was otherwise identical to the previously described version.

To investigate the impact of different assay readouts, we also performed electrochemiluminescence immunoassays (ECLIAs) using the Meso Scale Discovery (MSD) platform, as well as a bead-based assay using the Luminex Magplex platform. The MSD ECLIA was analogous to the site-specific ELISA described above: 100 μL/well of 0.5 μg/mL biotinylated antigen was coated onto MSD Gold streptavidin-coated plates (MSD) for 1 h at room temperature while shaking. 100 μL/well of 1:3600 serum samples were incubated for 1 h at room temperature while shaking. 100 μL/well of 0.5 μg/mL MH16-1 mouse-anti-human IgG (Sanquin; as used in ELISAs), conjugated with MSD Sulfo-Tag reagent using an NHS-ester conjugation kit (MSD) per manufacturer instructions, was incubated for 1 h at room temperature while shaking. 100 μL/well MSD Gold read buffer A (MSD) was added, and ECL signals were measured using an MSD Sector Imager 2400 A instrument (MSD). Luminex measurements were performed as described previously.^12^ Briefly, antigens were covalently coupled to Luminex Magplex beads using a two-step carbodiimide reaction according to manufacturer's protocol (Luminex). 50 μL/well of 1:10,000 serum samples were incubated with approximately 1000 antigen-coupled beads overnight at 4 °C. After washing, beads were incubated with goat-anti-human IgG-PE (Southern Biotech) for 2 h at room temperature while shaking. Afterwards, the beads were washed and measured on a MAGPIX reader instrument (Luminex) in 70 μL Magpix drive fluid (Luminex). Resulting MFI values are the median of approximately 50 beads per well, corrected by subtraction of MFI values from wells ran only with buffer and beads.

#### Competition ELISAs

To assess serum neutralization capacity, we performed an in-house developed and previously described competition ELISA.^10^ In short, the assay involves pre-incubation of sample serum with biotinylated RBD which is then added to immobilised angiotensin-converting enzyme 2 (ACE2); more potently neutralizing serum will leave less free RBD to bind ACE2 and generate signal. An additional BA.1 version of the assay was developed for this study using BA.1 RBD and 2022 pooled convalescent plasma as described above. Both WH1 and BA.1 versions of the assay were also adapted to a half-surface microwell plate, halving all working volumes (100 μL–50 μL) compared to the previously described protocol. Samples were measured at 8 serial dilutions each.

A selection of HC, DC and MTX monotherapy participants were tested in this assay; selections were made by ranking by anti-WH1 RBD titre at V3_post_ and selecting one random individual per 5-percentile interval within their group.

Data is presented as the percentage non-inhibited signal compared to the calibrator, where 100% indicates no inhibition and 0% indicates maximum inhibition achieved by the calibrator, plotted against the effective titre, which was calculated as the anti-WH1 RBD titre for WH1 RBD competition or anti-BA.1 RBD titre for BA.1 RBD competition divided by dilution factor.

Curves were fitted to each sample's measurements using the 4-parameter logistic model, constrained only so that the slope parameter should be <0 (decreasing signal with increasing effective titre). Samples from which no meaningful fit could be obtained were not considered for further analysis. IC_50_ and IC_80_ titres were calculated by solving the 4-parameter logistic equation $y=d+\frac{a-d}{1+{\left(\frac{x}{c}\right)}^{b}}$ for *x*, where *a* is the upper asymptote, *b* is the inflection point, *c* is the slope and *d* is the lower asymptote, using the fitted parameters with *y* = 50 and *y* = 20 respectively. Participants' fold decreases in IC_50_ and IC_80_ titres from V2_post_ to V3_post_ were calculated as $\frac{1}{\frac{{V3}_{\text{post}}{\;\text{IC}}_{50}\;\text{titre}}{{V2}_{\text{post}}{\;\text{IC}}_{50}\;\text{titre}}}$ and $\frac{1}{\frac{{V3}_{\text{post}}{\;\text{IC}}_{80}\;\text{titre}}{{V2}_{\text{post}}{\;\text{IC}}_{80}\;\text{titre}}}$ respectively.

#### Antibody validation statement

The above assays employ mouse anti-human IgG clone MH16-1 (all isotype-specific assays except WH1 RBD IgM ELISA) or mouse anti-human IgM clone MH15-1 (WH1 RBD IgM ELISA only). Both clones are commercially available, and have been validated and used for broad purposes including ELISA. The following RRIDs have been used to describe MH16-1: RRID:AB_672087, RRID:AB_419547, RRID:AB_1541678, RRID:AB_1002880, RRID:AB_10631952, RRID:AB_1286021 and RRID:AB_1075983. The following RRIDs have been used to describe MH15-1: RRID:AB_733098, RRID:AB_672095, RRID:AB_1542024, RRID:AB_1003785, RRID:AB_10633252 and RRID:AB_1076044.

### Statistics

Upper and lower bounds of 95% confidence intervals of medians were calculated through Conover's method for the estimation of population quantiles^13^: the positions in the sorted sample data of two observations whose values delineate the confidence interval is given by $np\;\pm \;z_{q}\sqrt{np\left(1-p\right)}$ rounded up to the next integer, where *n* is the sample size, *p* is the quantile of interest (0.5 for the median), and *z*_*q*_ is the z-value of the desired confidence level (1.96 for 95% confidence). The median and 95% confidence intervals of differences were calculated through Bauer's method, using the Hodges-Lehmann difference estimator.^14^

Titre ratios were analysed using the Kruskal–Wallis test and the Conover–Iman post-hoc multiple comparisons test with Benjamini-Hochberg (FDR) correction. For this primary analysis, to detect differences of Cohen's *d* of 0.5 at 80% power and multiple comparison-adjusted alpha of 0.05, we calculated a minimum sample size of N = 81 per group. Competition ELISA IC_50_ and IC_80_ titre values were analysed using Mann–Whitney U-tests. Analysis and visualization was performed using R version 4.1.2 with packages ‘tidyverse’ version 1.3.1, ‘rstatix’ version 0.7.0, ‘conover.test’ version 1.1.5, ‘pwrss’ version 0.3.1, ‘scales’ version 1.2.1, ‘dr4pl’ version 2.0.0, ‘ggpubr’ version 0.4.0 and ‘patchwork’ version 1.1.2.^15^^,^^16^

### Role of funders

The funders had no involvement in study design, data collection, analysis and interpretation, or the writing and submission for publication of this report.

## Results

### Participant characteristics

**Fig. 1:**
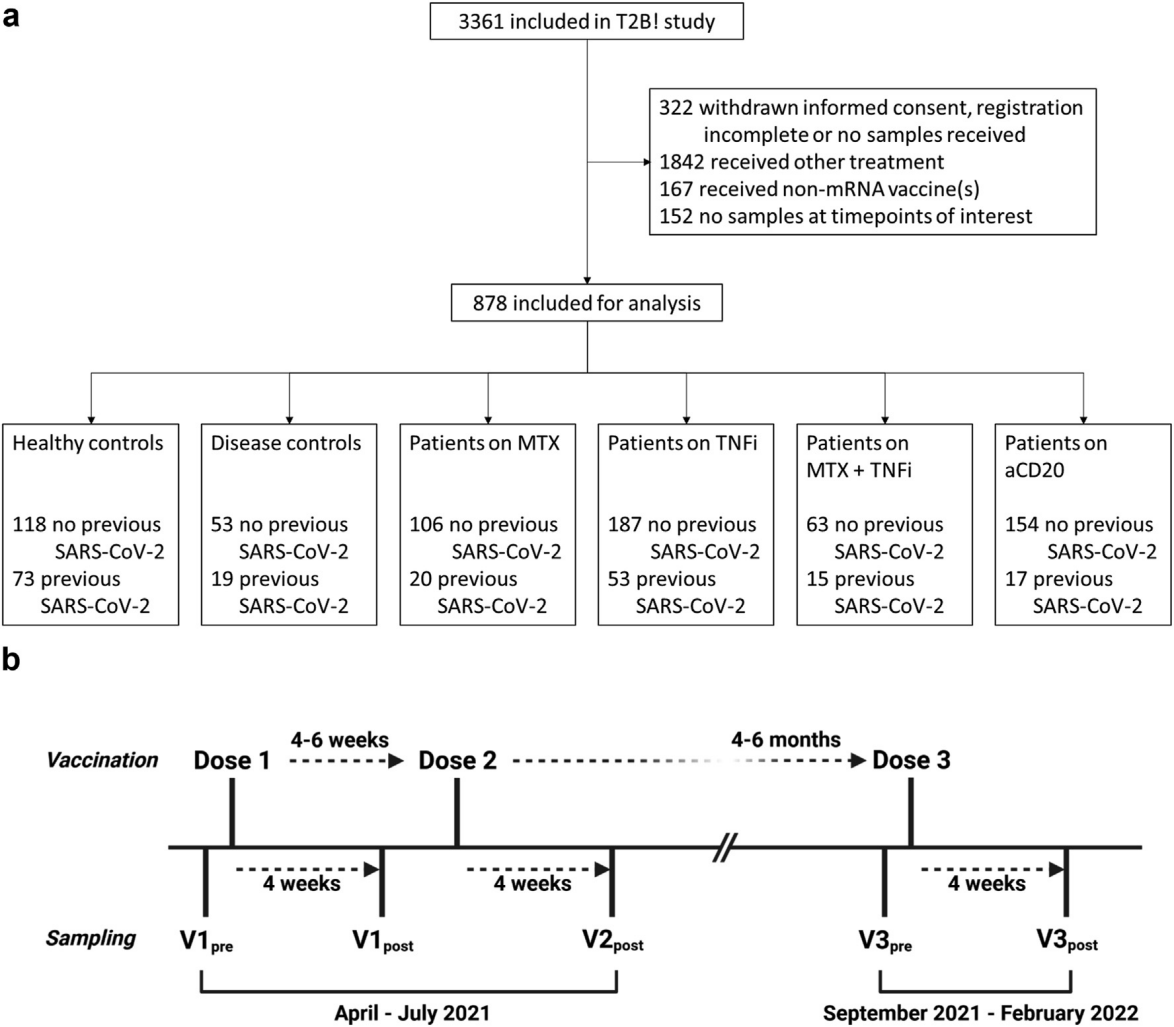
**Study overview**. **a** Study flowchart. **b** Vaccination and sampling timeline.

We included 878 participants from the ongoing Target-2-B! Immunity against SARS-CoV-2 vaccination cohort for analysis (Fig. 1a, Table 1): HC (N = 118 naïve and 73 antigen-experienced), DC (N = 53 and 19), MTX (N = 106 and 20), TNFi (N = 187 and 53), MTX + TNFi (N = 63 and 15) and anti-CD20 (N = 154 and 17). TNFi agents included mostly infliximab (N = 107, 45%), adalimumab (N = 80, 33%) and etanercept (N = 37, 15%) in the monotherapy group, and adalimumab (N = 40, 51%) and etanercept (N = 27, 35%) in the MTX combination therapy group; anti-CD20 agents were relatively even between ocrelizumab (N = 97, 55%) and rituximab (N = 80, 45 = %). The mean age of participants was 50.4 years (SD, 13.9); 63.3% of participants were female. The median interval between first and second vaccination was 36 days (IQR, 35–42); between second and third, 187 days (IQR, 149–198). An overview of vaccination and sampling timepoints is presented in Fig. 1b.

**Table 1:** Demographic and clinical characteristics of study participants.

	Overall (N = 878)	No prior SARS-CoV-2 infection						Prior SARS-CoV-2 infection					
		Healthy controls (N = 118)	Patients not on ISP (N = 53)	Patients on MTX (N = 106)	Patients on TNFi (N = 187)	Patients on MTX + TNFi (N = 63)	Patients on aCD20 (N = 154)	Healthy controls (N = 73)	Patients not on ISP (N = 19)	Patients on MTX (N = 20)	Patients on TNFi (N = 53)	Patients on MTX + TNFi (N = 15)	Patients on aCD20 (N = 17)
**Age (years), mean (SD)**	50.4 (13.9)	50.2 (10.5)	50.8 (14.0)	58.2 (13.3)	47.2 (14.6)	57.4 (14.0)	50.8 (13.8)	47.2 (12.4)	44.7 (13.2)	53.1 (13.8)	42.0 (13.2)	49.4 (8.86)	49.4 (13.5)
**Sex**													
Female	556 (63%)	77 (65%)	31 (58%)	80 (75%)	103 (55%)	44 (70%)	97 (63%)	45 (62%)	11 (58%)	12 (60%)	32 (60%)	12 (80%)	12 (70%)
Male	322 (37%)	41 (35%)	22 (42%)	26 (25%)	84 (45%)	19 (30%)	57 (37%)	28 (38%)	8 (42%)	8 (40%)	21 (40%)	3 (20%)	5 (30%)
**BMI (kg/m**^**2**^**), mean (SD)**	25.2 (4.9)	24.9 (4.3)	25.3 (5.2)	26.6 (5.7)	24.6 (5.0)	25.9 (5.2)	25.5 (5.2)	24.1 (3.3)	24.3 (4.2)	26.6 (4.8)	24.8 (4.4)	25.4 (4.6)	24.9 (4.4)
**Early third vaccination**													
Yes	445 (50%)	54 (46%)	19 (36%)	71 (67%)	73 (39%)	31 (49%)	136 (88%)	14 (19%)	3 (16%)	10 (50%)	14 (26%)	4 (27%)	16 (94%)
No	433 (50%)	64 (54%)	34 (64%)	35 (33%)	114 (61%)	32 (51%)	18 (12%)	59 (81%)	16 (84%)	10 (50%)	39 (74%)	11 (73%)	1 (6%)
**Vaccination interval (days), median (IQR)**													
V1–V2	36 (35–42)	36 (35–42)	36.5 (35–42)	36 (35–42)	35 (35–36.5)	36 (35–40.5)	36 (35–42)	42 (42–42)	36 (35–40)	35.5 (35–42)	36 (35–42)	36 (35–42)	36 (35–42)
V2–V3	187 (149–198)	190 (179–197)	200 (190–212)	190 (113–201)	192 (179–203)	194 (189–205)	125 (117–148)	190 (183–203)	175 (159–185)	189 (187–206)	191 (182–197)	195 (188–202)	150 (122–175)
**Additional ISP**													
Yes	44 (5%)	–	–	–	–	–	37 (24%)	–	–	–	–	–	7 (41%)
No	834 (95%)	118 (100%)	53 (100%)	106 (100%)	187 (100%)	63 (100%)	117 (76%)	73 (100%)	19 (100%)	20 (100%)	53 (100%)	15 (100%)	10 (59%)
**Rheumatic disorders**													
Rheumatoid arthritis	158 (18%)	–	1 (2%)	54 (51%)	22 (12%)	41 (65%)	11 (7%)	–	2 (11%)	10 (50%)	5 (9%)	10 (67%)	2 (12%)
Spondylarthritis	81 (9%)	–	1 (2%)	13 (12%)	40 (21%)	12 (19%)	–	–	–	2 (10%)	10 (19%)	3 (20%)	–
Systemic lupus erythematosus	9 (1%)	–	–	2 (2%)	–	–	5 (3%)	–	1 (5%)	1 (5%)	–	–	–
Vasculitis	33 (4%)	–	3 (6%)	1 (1%)	–	–	28 (18%)	–	–	–	–	–	1 (6%)
Other rheumatological	3 (<1%)	–	1 (2%)	1 (1%)	1 (1%)	–	–	–	–	–	–	–	–
**Inflammatory Bowel Disease**													
Crohn's disease	128 (15%)	–	6 (11%)	1 (1%)	91 (49%)	5 (8%)	–	–	2 (11%)	.	23 (43%)	–	–
Ulcerating colitis	82 (9%)	–	32 (60%)	–	32 (17%)	–	–	–	5 (26%)	.	13 (25%)	–	–
**Neurological disorders**													
Multiple sclerosis and neuromyelitis optica	106 (12%)	–	1 (2%)	–	–	–	91 (59%)	.	3 (16%)	–	–	–	11 (65%)
Inflammatory neuropathies and myopathies	15 (2%)	–	1 (2%)	4 (4%)	–	–	5 (3%)	–	1 (5%)	3 (15%)	–	–	1 (6%)
Myasthenia gravis	3 (<1%)	–	1 (2%)	1 (1%)	–	–	–	–	1 (5%)	.	–	–	–
**Dermatological disorders**													
Atopic dermatitis	4 (<1%)	–	–	3 (3%)	–	–	–	–	–	1 (5%)	–	–	–
Other dermatological	56 (6%)	–	5 (9%)	26 (25%)	1 (1%)	5 (8%)	7 (5%)	–	3 (16%)	3 (15%)	2 (4%)	2 (13%)	2 (12%)
**Other IMID**													
IgG4-related disease	1 (<1%)	–	–	–	–	–	1 (1%)	–	–	–	–	–	–

Data are presented as n (%) unless noted otherwise.

### Impact of epitope density on serological antibody measurements

**Fig. 2:**
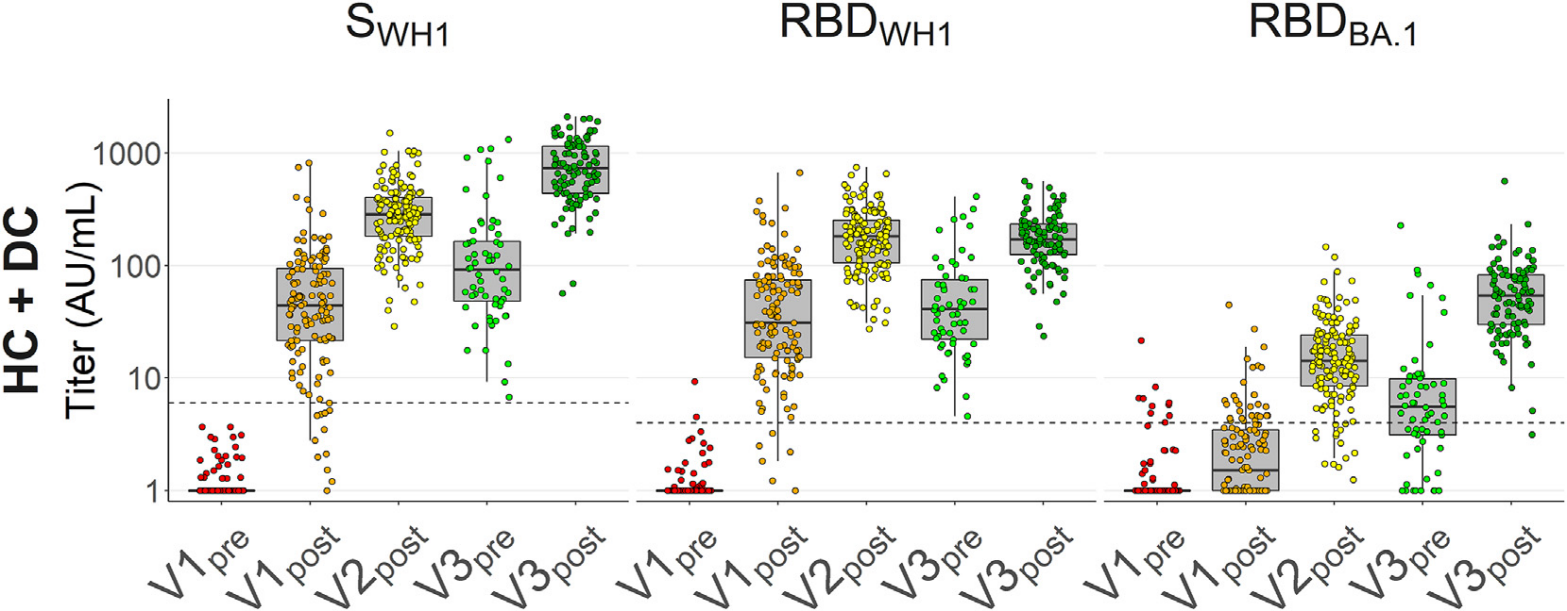
**Serum IgG titres against SARS-CoV-2 WH1 S, WH1 RBD, and BA.1 RBD in healthy and disease controls.** Serum IgG concentrations were assessed by direct ELISA and titres were calculated in arbitrary units (AU) derived from pooled convalescent healthy donor plasma standards collected in early 2020 (WH1 standard) or early 2022 (BA.1 standard), which were set at 100 AU/mL. WH1, Wuhan-Hu-1; RBD, receptor-binding domain; S, spike (full protein). Box plots show anti-WH1 S, anti-WH1 RBD, and anti-BA.1 RBD titres of controls (N = 118 healthy controls + N = 53 disease controls) without infection before vaccination. Dashed lines in titre plots represent seropositivity cutoffs determined as the lowest integer AU value where >99% of pre-pandemic samples were considered negative. In all box plots, central lines indicate the median, with hinges indicating 25th and 75th percentiles. Whiskers indicate the furthest data points up to 1.5 ∗ IQR beyond hinges. V1, V2, V3; first, second and third vaccination.

We initially sought to investigate changes in antibody repertoire by comparing longitudinal responses against WH1 S, WH1 RBD and BA.1 RBD (Fig. 2). We observed in HC and DC (“controls”, pooled for analysis) that anti-WH1 RBD titres did not increase from V2_post_ to V3_post_, whereas anti-WH1 S and BA.1 RBD did. Part of this change was also measurable at V3_pre_, indicating a shifting repertoire not only in response to a third vaccination but also as an ongoing effect over time. However, comparative measurements with different assay platforms found anti-WH1 RBD titres to also increase from V2_post_ to V3_post_, suggesting a major impact of the antigen immobilization method on observed antibody binding (Fig. S1a and b). We hypothesised epitope density to be a major factor responsible for these discrepancies. Indeed, varying the m/v protein coating concentration had a notable effect on the difference in measured IgG binding signal between V2_post_ and V3_post_ samples. This difference was most pronounced in the random immobilization RBD assay (Fig. S1c).

This can likely be explained by the fact that the RBD is a relatively small part of full-length S, and immobilizing the same m/v concentration yields a considerably greater molar concentration of RBD than S, thus higher epitope density. Particularly, it appears that a critical point where epitope density becomes high enough to allow binding with both Fab arms per antibody is reached for RBD but not for full-length S in this range of commonly used m/v coating concentrations for ELISA. The RBD assay therefore represents mostly a measurement of concentration that is relatively insensitive to changes in affinity, as binding with both Fab arms will grant most antibodies sufficient avidity to produce assay signal even with low affinity per Fab arm. Conversely, the full S protein assay will reflect single-Fab binding affinity to a substantial degree, and changes in affinity over time may be captured alongside changes in epitope preference when comparing S and RBD data. The comparison of WH1 and BA.1 RBDs at equally high density should reveal cross-reactivity without these confounding effects.

### Increasing BA.1 cross-reactivity after repeat vaccination and previous infection

**Fig. 3:**
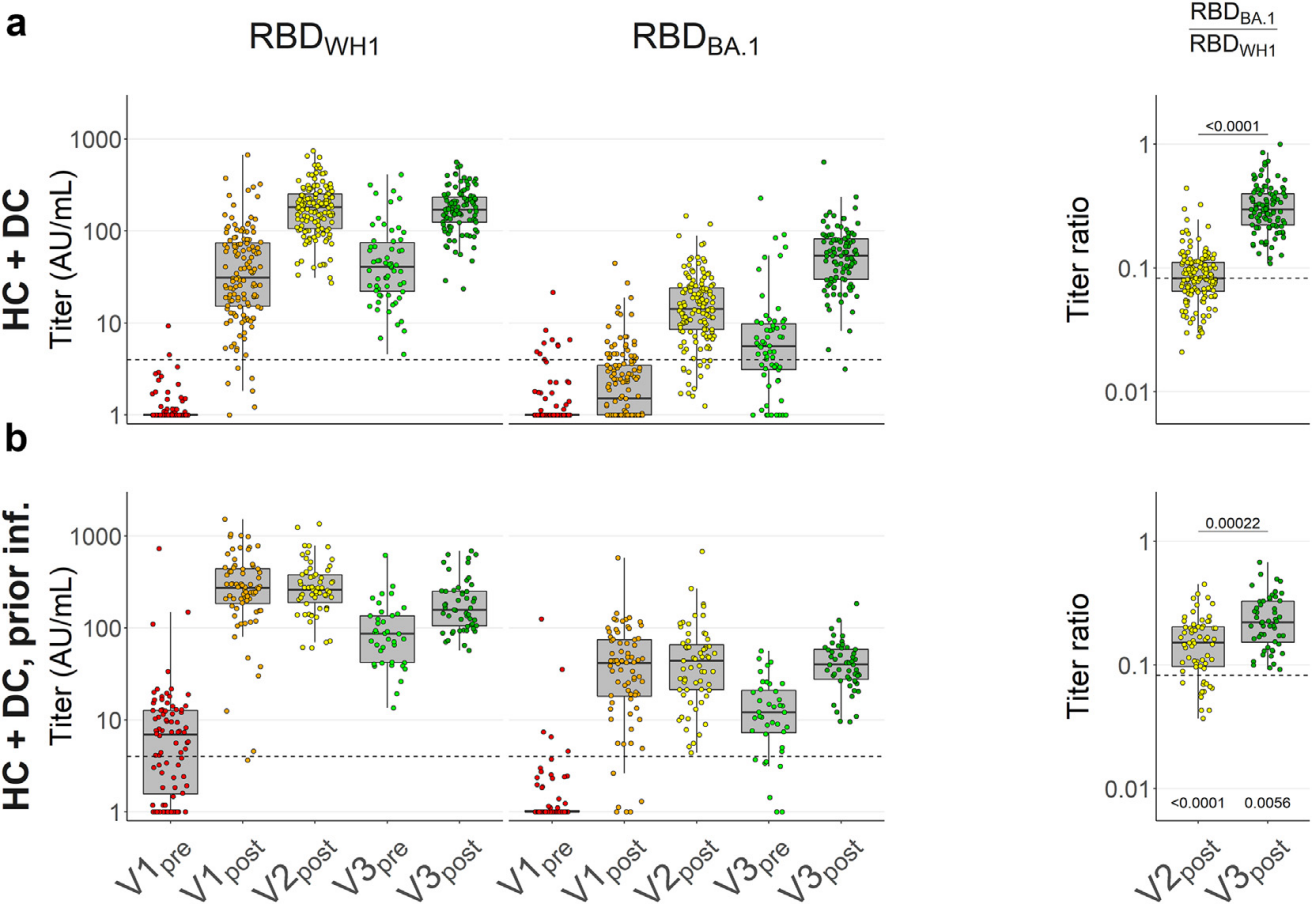
**Serum IgG titres against SARS-CoV-2 WH1 and BA.1 RBD, WH1 S and BA.1 RBD in healthy and disease controls with and without prior infection**. Serum IgG concentrations were assessed by direct ELISA and titres were calculated in arbitrary units (AU) derived from pooled convalescent healthy donor plasma standards collected in early 2020 (WH1 standard) or early 2022 (BA.1 standard), which were set at 100 AU/mL. WH1, Wuhan-Hu-1; RBD, receptor-binding domain. **a and b** Box plots showing anti-WH1 RBD and anti-BA.1 RBD titres, and comparative ratios of controls without infection before vaccination (**a**, N = 118 healthy controls + N = 53 disease controls, titre data repeated from Fig. 2) and with prior infection (**b**, N = 73 + 19). Dashed lines in titre plots represent seropositivity cutoffs determined as the lowest integer AU value where >99% of pre-pandemic samples were considered negative. Dashed lines in ratio figures represent the median of naïve controls (**a**) at V2_post_ for comparison. In all box plots, central lines indicate the median, with hinges indicating 25th and 75th percentiles. Whiskers indicate the furthest data points up to 1.5 ∗ IQR beyond hinges. V1, V2, V3; first, second and third vaccination. Significance marks below box plots indicate comparisons to naïve controls (**a**) at the same timepoint. Comparisons were made using Kruskal–Wallis tests and Conover–Iman post-hoc multiple comparisons with Benjamini-Hochberg correction.

To explore changes in cross-reactivity, we determined IgG titres against the WH1 and Omicron BA.1 RBDs and compared ratios thereof. In naïve controls, we found that BA.1 RBD-binding IgG was detectable in a majority of individuals at V2_post_, decayed but remained above V1_post_ levels at V3_pre_, and increased significantly [Conover–Iman test] from the V2_post_ peak at V3_post_. Median ratios of anti-BA.1 RBD/WH1 RBD increased significantly from 0.08 at V2_post_ to 0.30 at V3_post_, while low V3_pre_ anti-BA.1 RBD titres precluded reliable evaluation of ratios for that time point. These trends were comparable when HC and DC were analysed separately (Fig. S2). We did not observe major differences in participants who experienced a breakthrough infection after V2_post_ other than generally increased titres at V3_pre_, presumably resulting simply from this more recent recall response (Fig. 3a). Descriptive statistics by timepoint are presented in Table 2. Taken together, the proportionally enhanced response against BA.1 RBD after third vaccination reflects a shifting antibody repertoire that overall becomes better able to bind the mutated BA.1 RBD, likely by increased targeting of conserved RBD epitopes as the majority of individuals remained naïve to the mutated BA.1 epitopes at the time of the third vaccination.

**Table 2:** Overview of IgG titre ratios.

Group	Ratio RBD_BA.1_/RBD_WH1_			
	V2_post_	Difference from HC + DC	V3_post_	Difference from HC + DC
HC + DC	**0.08** (0.06–0.11) N = 144	–	**0.30** (0.22–0.39) N = 103	–
HC + DC, prior infection	**0.15** (0.10–0.20) N = 63	**0.061** (0.039–0.082)	**0.22** (0.15–0.33) N = 52	**−0.072** (−0.114 to −0.034)
MTX	**0.09** (0.06–0.17) N = 100	**0.015** (0.001–0.031)	**0.42** (0.28–0.57) N = 77	**0.111** (0.054–0.171)
TNFi	**0.10** (0.06–0.16) N = 168	**0.02** (0.008–0.032)	**0.24** (0.16–0.34) N = 109	**−0.062** (−0.098 to −0.026)
MTX + TNFi	**0.09** (0.06–0.18) N = 59	**0.012** (−0.005 to 0.034)	**0.25** (0.12–0.32) N = 41	**−0.074** (−0.126 to −0.022)

Descriptive statistics supplemental to Figs. 3 and 5. Data are presented as median (IQR) and N.

We also considered whether a previous infection might lead to altered dynamics. Therefore, we analysed the responses of controls who experienced an infection prior to vaccination (Fig. 3b). As expected from previous antigen exposure and a subsequent recall response, WH1 RBD titres at V1_pre_ as well as V1_post_ were greatly elevated compared to those of naïve individuals, and less so at V2_post_. Notably, antigen-experienced individuals retained considerably higher titres by V3_pre_, and no further increase from V2_post_ to V3_post_ was observed. With respect to anti-BA.1 RBD titres, we found an increased median ratio of 0.15 at V2_post_, while a much less steep rise at V3_post_ resulted in a median ratio of 0.22, which was significantly lower compared to naïve individuals at the same timepoint, mostly due to lower anti-BA.1 RBD titres in the antigen-experienced group.

**Fig. 4:**
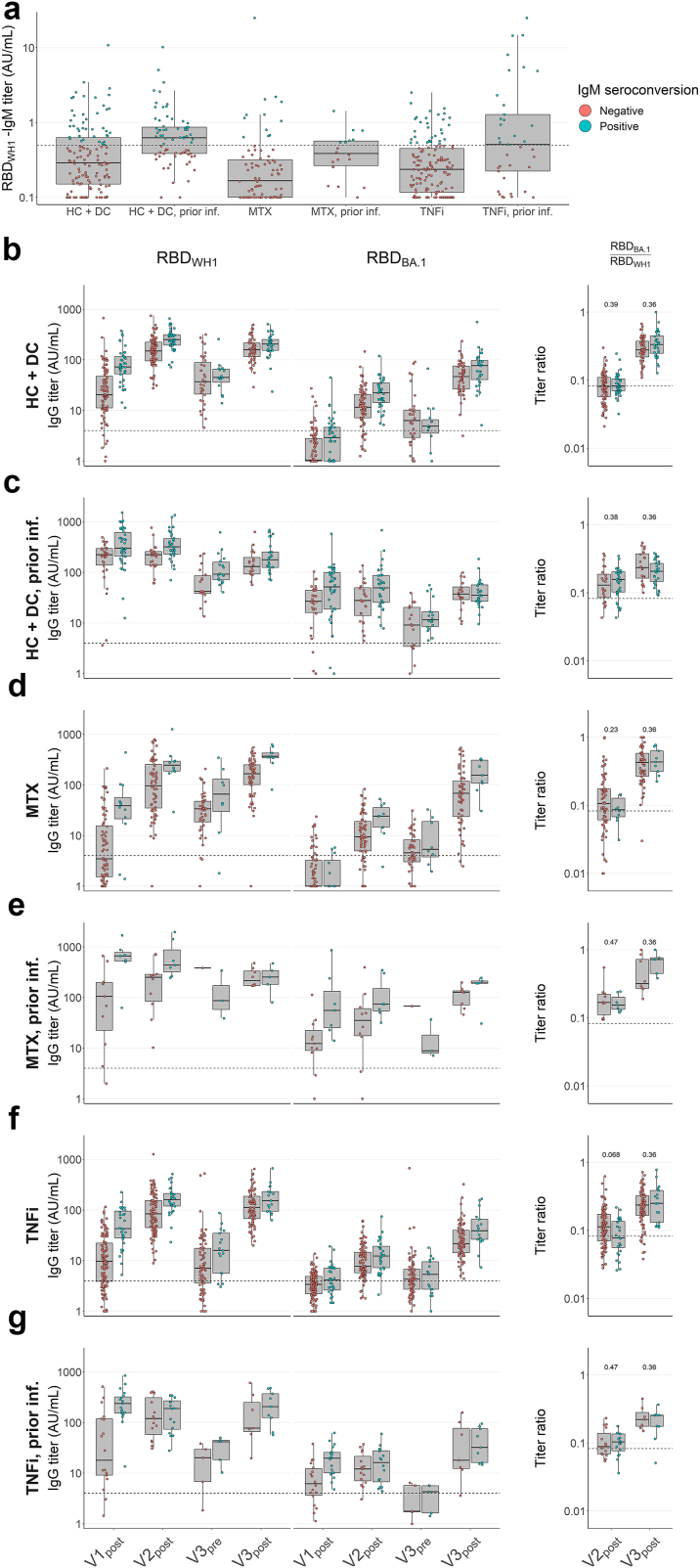
**Serum IgG titres against SARS-CoV-2 WH1 and BA.1 RBD, WH1 S and BA.1 RBD in healthy and disease controls, and patients under immunosuppressive treatment, with and without prior infection, as predicted by IgM titres after first vaccination**. Serum IgG and IgM concentrations were assessed by direct ELISA and titres were calculated in arbitrary units (AU) derived from pooled convalescent healthy donor plasma standards collected in early 2020 (WH1 standard) or early 2022 (BA.1 standard), which were set at 100 AU/mL. WH1, Wuhan-Hu-1; RBD, receptor-binding domain. **a** Box plots showing anti-WH1 RBD IgM titres at V1_post_. **b–g** Box plots showing anti-WH1 RBD and anti-BA.1 RBD IgG titres, and comparative ratios, divided and compared by IgM seroconversion as shown in (**a**). Panel **b** corresponds to data shown in Fig. 3a–d to Fig. 5a, b e, f and g. Dashed lines in titre plots represent seropositivity cutoffs determined as the lowest integer AU value where >99% of pre-pandemic samples were considered negative. Dashed lines in ratio figures represent the median of naïve controls (Fig. 2a) at V2_post_ for comparison. In all box plots, central lines indicate the median, with hinges indicating 25th and 75th percentiles. Whiskers indicate the furthest data points up to 1.5 ∗ IQR beyond hinges. V1, V2, V3; first, second and third vaccination. Significance marks above box plots indicate comparisons between V1_post_ IgM-negative and -positive subgroups. Comparisons were made using Kruskal–Wallis tests and Conover–Iman post-hoc multiple comparisons with Benjamini-Hochberg correction.

Following from this, we sought to investigate whether a more robust early response could predict greater cross-reactivity. To this end, we determined anti-WH1 RBD-IgM titres at V1_post_ and compared longitudinal IgG response data with the additional grouping of early IgM responders and non-responders (Fig. 4). We observed generally higher IgG titres in the IgM-positive groups of both naïve and previously infected controls (Fig. 4b and c), particularly at early timepoints. However, titre ratios did not significantly differ between positive and negative individuals, suggesting that a more robust early IgM response does not predict a more cross-reactive long-term IgG response. Taken together, these data indicate that individuals who experienced an early SARS-CoV-2 infection already underwent a degree of repertoire evolution before vaccination, with naïve individuals converging to a similar state after three vaccine doses, and that the magnitude of the early (IgM) response does not greatly affect the development of a cross-reactive response.

### Repertoire evolution is qualitatively similar in patients treated with MTX- and/or TNFi but absent with anti-CD20

**Fig. 5:**
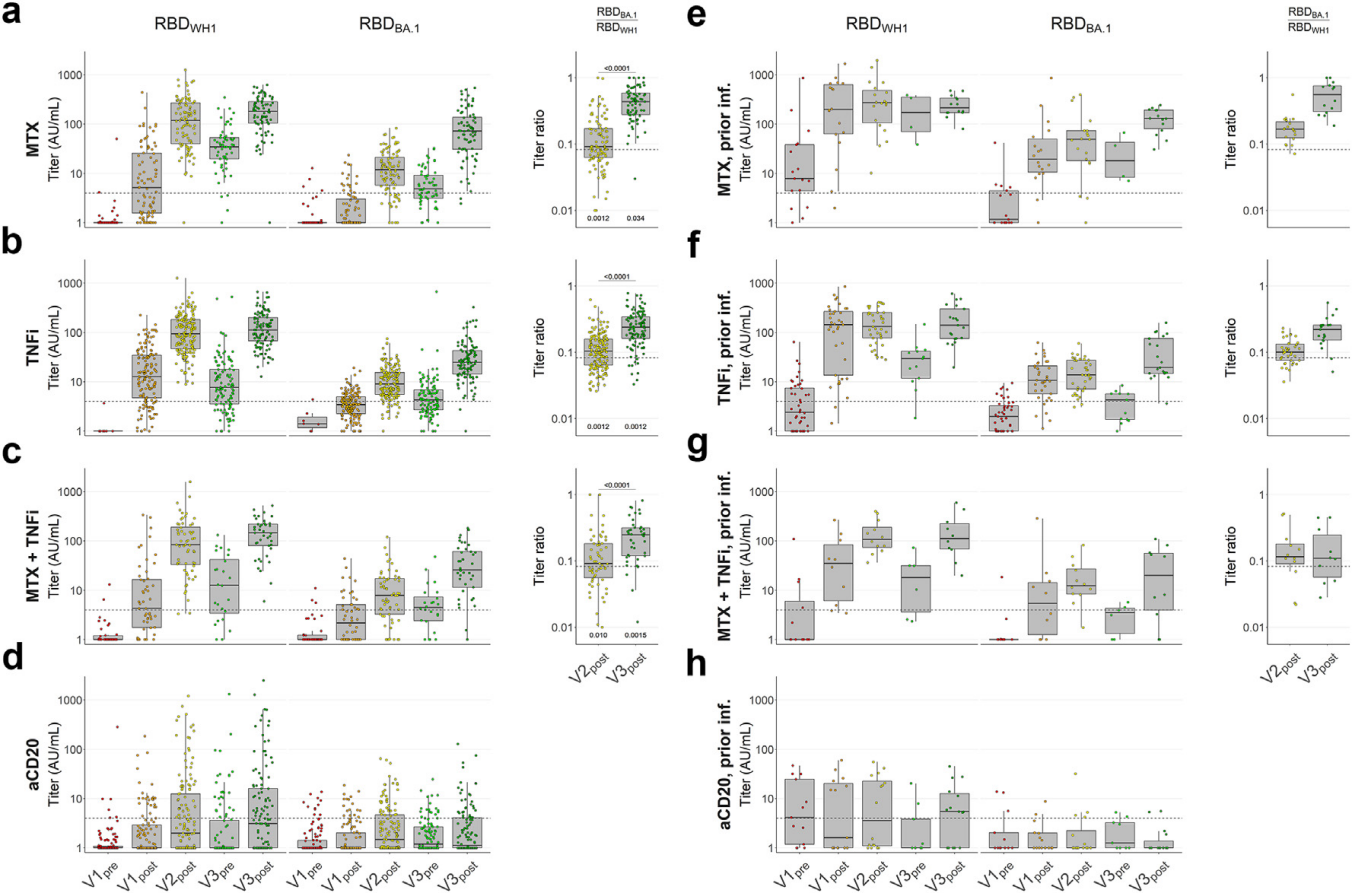
**Serum IgG titres against SARS-CoV-2 WH1 and BA.1 RBD, WH1 S and BA.1 RBD in patients under immunosuppressive treatment with and without prior infection**. Serum IgG concentrations were assessed by direct ELISA and titres were calculated in arbitrary units (AU) derived from pooled convalescent healthy donor plasma standards collected in early 2020 (WH1 standard) or early 2022 (BA.1 standard), which were set at 100 AU/mL. WH1, Wuhan-Hu-1; RBD, receptor-binding domain; S, spike (full protein). **a–h** Box plots showing anti-WH1 RBD and anti-BA.1 RBD titres, and comparative ratios of patients without infection before vaccination on MTX monotherapy (**a**, N = 106), patients on TNFi monotherapy (**b**, N = 187), patients on MTX + TNFi combination therapy (**c**, N = 63), patients on anti-CD20 therapy (**d**, N = 154), and the same groups with prior infection (**e–h**, N = 20, 53, 15, and 17 respectively). Dashed lines in titre plots represent seropositivity cutoffs determined as the lowest integer AU value where >99% of pre-pandemic samples were considered negative. Dashed lines in ratio figures represent the median of naïve controls (Fig. 3a) at V2_post_ for comparison. In all box plots, central lines indicate the median, with hinges indicating 25th and 75th percentiles. Whiskers indicate the furthest data points up to 1.5 ∗ IQR beyond hinges. V1, V2, V3; first, second and third vaccination. Significance marks below box plots indicate comparisons to naïve controls (Fig. 3a) at the same timepoint. Comparisons were made using Kruskal–Wallis tests and Conover–Iman post-hoc multiple comparisons with Benjamini-Hochberg correction.

We next examined the effects of immunosuppressive agents. SARS-CoV-2 naïve patients on MTX monotherapy exhibited moderately diminished anti-WH1 RBD titres up to V2_post_ compared to the control groups, but retained similar titres at V3_pre_ and saw a modest peak-to-peak rise at V3_post_ (Fig. 5a). Anti-BA.1 RBD titres also emerged with kinetics comparable to controls (ratios of 0.09 and 0.42, at V2_post_ and V3_post_ respectively). Patients on TNFi monotherapy showed similarly diminished anti-WH1 RBD titres compared to controls, with notably greater decay at V3_pre_ and particularly low anti-BA.1 RBD titres at V3_post_ (Fig. 5b). Anti-BA.1 RBD/WH1 RBD ratio increased from 0.10 at V2_post_ to 0.23 at V3_post_. The effects of both drugs were also evident in patients receiving MTX + TNFi combination therapy, including reduced peak titres at V1_post_ and V2_post_, and greater decay at V3_pre_ respectively (Fig. 5c), though the overall trend of repertoire evolution was again similar to controls. Descriptive statistics by timepoint are presented in Table 2. Conversely, in patients on anti-CD20 treatment, we found only minor to no cumulative effects from repeated vaccination. Titres remained heavily diminished over time, with approximately 50% of individuals below anti-WH1 RBD titre cutoffs at all timepoints, and as many as 75% below the anti-BA.1 RBD titre cutoff despite three vaccinations (Fig. 3d). Across MTX, TNFi and combination therapy groups, patients who experienced a SARS-CoV-2 infection prior to vaccination exhibited similar trends compared to antigen-experienced controls (Fig. 5e–g), though a third vaccination was required to induce similarly high anti-BA.1 RBD titres, while patients on anti-CD20 treatment were still broadly unable to develop anti-BA.1 RBD responses (Fig. 5h). Furthermore, as in controls, we found no significant differences in titre ratios between early IgM-positive and -negative patients on MTX and TNFi monotherapies (Fig. 4d–g). Taken together, these data indicate that three vaccinations induce a shift in the antibody repertoire in patients on moderate immunosuppression by MTX and/or TNFi similar to controls, but not in patients on anti-CD20 treatment.

### RBD-ACE2-binding interference improves as part of repertoire evolution after the third vaccination

**Fig. 6:**
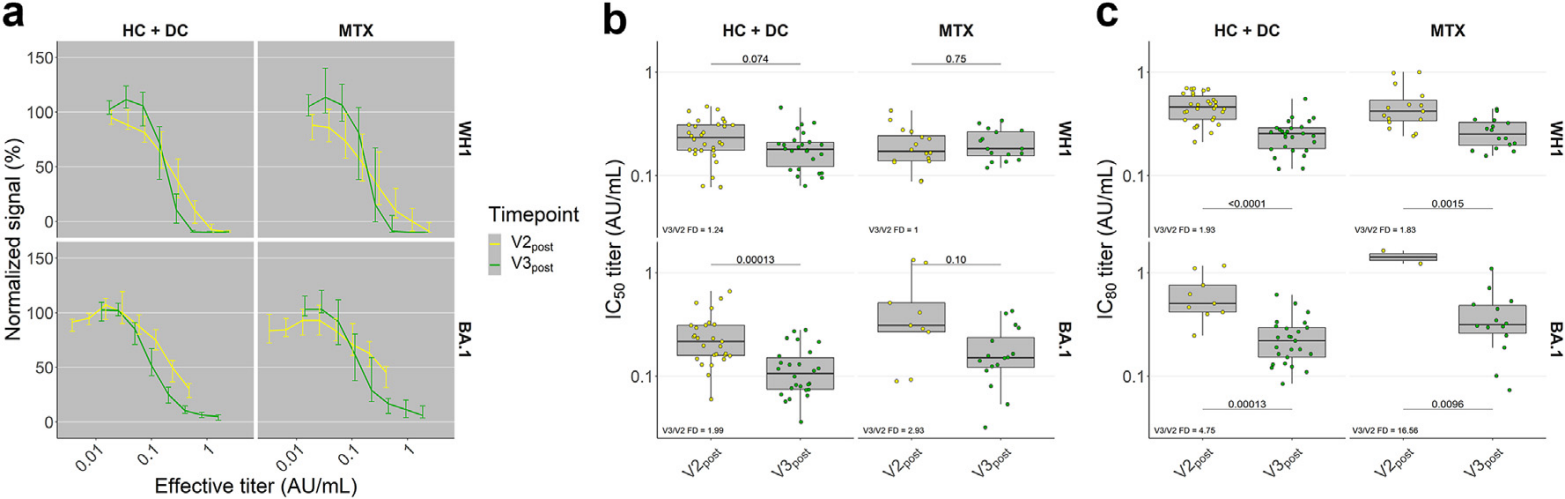
**Serum RBD-ACE2 binding inhibition**. Serum capacity to interfere with RBD-ACE2 binding was assessed by competition ELISA. RBD, receptor-binding domain of spike protein; ACE2, human angiotensin-converting enzyme 2. Inhibition was expressed as remaining signal intensity normalised to that of pooled convalescent healthy donor plasma standards collected in early 2020 (WH1 standard) or early 2022 (BA.1 standard), where 100% represents signal intensity at the lowest concentration of standard and 0% represents signal intensity at the highest concentration of standard. WH1, Wuhan-Hu-1. **a** Line plots showing inhibition as a function of effective titre (corresponding anti-WH1 or anti-BA.1 RBD titre divided by dilution factor) in N = 20 each healthy controls, disease controls (pooled as controls) and patients on MTX therapy. Curves presented as median normalised remaining signal with 95% confidence interval, and median effective titre. **b and c** Box plots showing individual IC50 (**b**) and IC80 (**c**) effective titres as calculated from 4-parameter logistic curve-fit. IC50, effective titre at which normalised signal was reduced to 50%; IC80, effective titre at which normalised signal was reduced to 20% (i.e. 80% inhibition). Samples are not shown in respective plots if IC50 or IC80 titre could not be derived through curve-fitting as in (**a**). In all box plots, central lines indicate the median, with hinges indicating 25th and 75th percentiles. Whiskers indicate the furthest data points up to 1.5 ∗ IQR beyond hinges. V1, V2, V3; first, second and third vaccination. Comparisons were made using Mann–Whitney U-tests and Benjamini-Hochberg correction. FD, fold decrease from V2post to V3post (median of individual fold decreases).

To gain further insight into the nature of repertoire evolution, we tested a selection of V2_post_ and V3_post_ samples from HC, DC and patients on MTX (N = 20 each) in a previously described competition assay^10^; HC and DC were pooled for analysis (controls). Unlike the immobilised setting of the titre-determining direct IgG ELISA assay format, this assay involves pre-incubation of serum with RBD in solution to directly interfere with subsequent RBD-ACE2 binding, and thus places greater emphasis on affinity. As inhibitory potency will still be determined by antibody concentration as well, we compared inhibition against dilution-adjusted anti-WH1 or anti-BA.1 RBD titres to focus on affinity effects (Fig. 6a). Overall, inhibition of WH1 RBD appeared to be only modestly affected between V2_post_ and V3_post_ both for controls and patients on MTX, with little change in IC_50_ in either group but significant 1.93 and 1.83-fold decreases in IC_80_ respectively (Fig. 6b and c); the latter resulted from steeper titration curves at V3_post_, which likely represents enhanced affinities. Inhibition curves against BA.1 RBD ran more parallel for controls, with significant decreases in both IC_50_ and IC_80_ (1.99 and 4.75 fold respectively) suggesting a more distinct difference in affinities from V2_post_ to V3_post_. This was particularly clear for MTX-treated patients, many of whose sera failed to achieve 50% and particularly 80% inhibition at V2_post_ despite most individuals having detectable anti-BA.1 RBD titres at that time (Fig. 5a). Inhibition improved greatly at V3_post_, with a 2.93-fold decrease in IC_50_, though lacking inhibitory performance from most samples at V2_post_ precluded reliable comparison of IC_80_. Taken together, these data point to affinity maturation as a key component of repertoire evolution both in controls and in MTX-treated IMID patients.

## Discussion

In summary, we show a clear emergence of anti-BA.1 RBD reactivity in patients treated with MTX and/or TNFi, but not in patients on anti-CD20 treatment. A previous WH1 infection before vaccination resulted in a much higher degree of anti-BA.1 RBD reactivity at early timepoints, with many individuals showing titres at V1_post_ or V2_post_ comparable to those of infection-naïve counterparts at V3_post_. Lastly, we find that inhibition of RBD-ACE2 binding against WH1 and BA.1 RBD after third vaccination appears to improve in part due to enhanced affinity. The responses that we detect at the serum level align with previous reports demonstrating that enhanced cross-reactivity to Omicron-lineage variants results from ongoing somatic hypermutation of clones already abundant shortly after second vaccination,^5^ particularly those that prefer more conserved epitopes of the RBD. Our data help to extend these previous observations to a substantially larger and more varied study population, and demonstrate that the widely used immunosuppressants MTX and TNFi do not fundamentally alter this repertoire evolution.

Fundamental mechanisms of these consistent changes in epitope preference have been investigated by others. Recent evidence points to epitope availability in germinal centres as the key mechanism driving this shift; as previously generated high-affinity antibodies bind their target epitope, memory B cells of the same specificity are blocked from acquiring the antigen, and naïve B cells of different specificities are instead able to bind and become activated,^17^ favouring specificities directed away from the RBD which dominates the initial response.^18^ This effect is strongest when the antigen triggering the recall response is identical or highly similar to the antigen that triggered the initial response,^19^ and has been suggested as an explanation to the phenomenon of original antigenic sin.^20^, ^21^, ^22^ Interestingly, though Yang et al. suggest that the increased germinal centre epitope availability driving the development of cross-reactivity at the time of a recall response is mediated in part by the enhanced trafficking of antigen by antibodies raised in the initial response forming immune complexes,^17^ and IgM-immune complex-driven loading of follicular dendritic cells has been described in mice,^23^ we found no significant differences in the development of cross-reactivity between individuals with a lacking or robust early IgM response, which is in agreement with the findings of Piubelli and Ruggiero et al. who found that strong early IgM responses predicted higher IgG titres over time but no qualitative differences.^24^

The interval between antigen challenges is also important in this regard; a previous study by Graham et al. found enhanced cross-reactivity after second vaccination when the second dose was given with a longer interval,^25^ in agreement with our data showing strong induction of anti-BA.1 RBD titres after only one dose in individuals that experienced a WH1 SARS-CoV-2 infection often months ahead of vaccination. Furthermore, while our data does not directly show shifting epitope preference within the RBD itself through comparison of anti-WH1 RBD and BA.1 RBD titres, this conclusion is in line with the functional improvement in RBD-ACE2 binding inhibition that we find after third vaccination. The contribution to cross-reactivity of increased affinity alone should therefore not be discounted.

It is thus reassuring that we find broadly similar patterns in patients treated with MTX and/or TNFi, in line with previous work showing comparable rates of breakthrough infections and disease severity.^26^ In case of TNFi, the proportion of antibodies binding to non-RBD parts is smaller at all time points in comparison to the control groups, but a skewing upon successive vaccinations towards more non-RBD reactivity is observed nonetheless. Numerous studies have investigated vaccination responses in IMID patients using these immunosuppressants, but relatively little longitudinal data exists in the literature.^27^ The mechanisms by which MTX and TNFi affect humoural responses after vaccination and infection also remain incompletely understood.^28^ TNFi has previously been reported to cause faster decay of humoural responses, but while it is thought to interfere with follicular dendritic cell function and germinal centre formation, effects on memory B cells have not been found.^29^, ^30^, ^31^ In contrast, we found long-term responses to be profoundly disrupted in patients on anti-CD20 treatment. We previously showed that there is a window during the standard 6-month dosing interval of ocrelizumab in which mRNA vaccination could elicit a relatively robust anti-WH1 RBD response.^32^ Our current results show that anti-WH1 RBD seropositive individuals at V2_post_ exhibit high spread in subsequent titres at V3_post_, and median anti-BA.1 RBD titres in particular fell. As such, repertoire evolution as seen in other groups could not generally be said to occur in patients treated with anti-CD20.

The main strength of this study is its large, disease-overarching cohort and further longitudinal analysis than previous studies investigating immunosuppressant effects on vaccination responses for several commonly used drugs, together with an internal control arm and assessment of hybrid immunity in antigen-experienced groups. We also characterise responses beyond magnitude alone to provide new insights in the nature of repertoire evolution and factors that affect it. The main limitation of this study is the use of serological assays as proxies to investigate processes considered to occur mainly in lymph node germinal centres, direct study of which was not possible with the fingerprick serum sampling used here. With regards to analysis, varying availability of samples across timepoints necessitated unpaired statistical testing, though sample sizes were sufficient to offset this power loss in all groups without prior infection other than patients treated with MTX and TNFi combination therapy. We did not consider dose effects within groups of the same immunosuppressants.

Overall, our data indicates that despite initially diminished titres or greater long-term decay, overall qualitative evolution of the antibody repertoire was minimally impacted by MTX and/or TNFi treatment, suggesting that fundamental mechanisms guiding this evolution were not critically impaired. Thus, while the direction of repertoire evolution and cross-variant protection will depend on both the immunogenicity and conservedness of different epitopes in a given antigen, patients treated with these immunosuppressants likely respond to vaccination in a manner comparable to healthy individuals in the long term, whereas patients treated with anti-CD20 do not.

## Contributors

**JBDK**: Methodology, Investigation, Validation, Data curation (serological assays), Visualization, Formal analysis, Writing—original draft; **EWS**: Data curation (clinical information), Writing—review & editing; **LW**: Data curation (clinical information), Writing—review & editing; **MS**: Investigation, Writing—review & editing; **KPJvD**: Data curation (clinical information), Writing—review & editing; **LYLK**: Data curation (clinical information), Writing—review & editing; **ZLEvK**: Resources (patients), Writing—review & editing; **JK**: Resources (patients), Writing—review & editing; **AGV**: Resources (patients), Writing—review & editing; **SWT**: Resources (patients), Writing—review & editing; **LB**: Resources (patients), Writing—review & editing; **GJW**: Resources (patients), Writing—review & editing; **LFB**: Writing—review & editing; **NJMV**: Writing—review & editing; **SK**: Investigation, Writing—review & editing; **GvM**: Investigation, Writing—review & editing; **OC**: Investigation, Writing—review & editing; **AJB**: Investigation, Writing—review & editing; **KvdS**: Investigation, Writing—review & editing; **JvR**: Investigation, Writing—review & editing; **MJvG**: Investigation, Writing—review & editing; **AtB**: Conceptualisation, Supervision, Writing—review & editing; **SMvH**: Conceptualisation, Supervision, Funding, Writing—review & editing; **TWK**: Conceptualisation, Supervision, Funding, Resources (patients), Writing—review & editing; **FE**: Conceptualisation, Supervision, Funding, Resources (patients), Writing—review & editing; **TR**: Conceptualisation, Supervision, Funding, Methodology, Investigation, Validation, Writing—review & editing. All authors read and approved the final version of the manuscript. T2B! study group members provided further assistance with patient recruitment and contact, as well as communication and logistics between study centres.

## Data sharing statement

After publication, anonymised individual data and a data dictionary will be made available under a data sharing agreement to researchers who provide a methodologically sound proposal to the corresponding author.

## Declaration of interests

For funding of this study, SMvH, FE, TWK and TR report grants from ZonMw and SMvH reports a grant from SGF to study immune responses after SARS-Cov-2 vaccination in autoimmune diseases. JK reports grants for multicentre investigator-initiated trials from ZonMw and Treatmeds; contracted research grants to his institution from F. Hoffmann-La Roche Ltd, Biogen, Immunic, Teva, Merck, Novartis and Sanofi/Genzyme; speaker's fees paid to his institution from F. Hoffmann-La Roche Ltd, Biogen, Immunic, Teva, Merck, Novartis and Sanofi/Genzyme; and compensation paid to his institution for participation in an adjudication committee of an MS clinical trial by Immunic. SWT reports grants paid to his institution from GlaxoSmithKline, Pfizer, Roche, AstraZeneca and Galapagos; and speaker's fees paid to his institution from NovoNordisk, AbbVie and UCB. SMvH reports compensation paid to her institution for participation in EU member expert panel HORIZON-HLTH-2023-DISEASE-03-18 and in the supervisory board of EU consortium INsTRuCT, from EU Horizon 2020 grants; unpaid participation in the supervisory board of national consortium DC4BALANCE, in the board of the European Federation of Immunological Societies, as co-project leader of ZonMw COVID-19 research program “Immunity against SARS-CoV-2 in immune-suppressed patients: increased risk of insufficient immunological memory or sufficient protection against re-infection?”, and in the project management board of national consortium ImmuneHealthSeed. FE reports grants from Prinses Beatrix Spierfonds, CSL Behring, Kedrion, Terumo BCT, Grifols, Takeda Pharmaceutical Company, and GBS-CIDP Foundation; consulting fees from UCB Pharma and CSL Behring; and honoraria from Grifols. All other authors declare no competing interests.
