# Supplementary material for: Evolution of SARS-CoV-2 antibody repertoire after successive mRNA vaccinations under immunosuppressive treatment
Source: eBioMedicine. 2025 Feb 25;113:105620. doi: 10.1016/j.ebiom.2025.105620 (PMC11905820; doi:10.1016/j.ebiom.2025.105620)
Supplement: Supplementary material [file mmc1.docx]

Supplementary material

“Evolution of SARS-CoV-2 antibody repertoire after successive mRNA vaccinations under immunosuppressive treatment”, Keijser *et al.* 2024, eBioMedicine

List of *T2B! Immunity against SARS-CoV-2* study group collaborators 2

List of T2B! Immunity against SARS-CoV-2 study group collaborators

| **First names** | **Surnames** |
| --- | --- |
| Anneke J. | Van der Kooi |
| Joop | Raaphorst |
| Mark | Löwenberg |
| R. Bart | Takkenberg |
| Geert R.A.M. | D'Haens |
| Phyllis I. | Spuls |
| Marcel W. | Bekkenk |
| Annelie H. | Musters |
| Nicoline F. | Post |
| Angela L. | Bosma |
| Marc L. | Hilhorst |
| Yosta | Vegting |
| Frederike J. | Bemelman |
| Alexandre E. | Voskuyl |
| Bo | Broens |
| Agner R. | Parra Sanchez |
| Cécile A.C.M. | Van Els |
| Jelle | De Wit |
| Abraham | Rutgers |
| Karina | De Leeuw |
| Barbara | Horváth |
| Jan J.G.M. | Verschuuren |
| Annabel M. | Ruiter |
| Lotte | Van Ouwerkerk |
| Diane | Van der Woude |
| Renée C.F. | Van Allaart |
| Y.K. Onno | Teng |
| Pieter | Van Paassen |
| Matthias H. | Busch |
| Papay B.P. | Jallah |
| Esther | Brusse |
| Pieter A. | Van Doorn |
| Adája E. | Baars |
| Dirk Jan | Hijnen |
| Corine R.G. | Schreurs |
| W. Ludo | Van der Pol |
| H. Stephan | Goedee |
| Koos A.H. | Zwinderman |
| Rivka | De Jongh |
| Carolien E. | Van de Sandt |
| Lisan H. | Kuijper |
| Mariël C. | Duurland |
| Ruth R. | Hagen |
| Jet | Van den Dijssel |
| Christine | Kreher |
| Amélie V. | Bos |
| Virginia | Palomares Cabeza |
| Veronique A.L. | Konijn |
| George | Elias |
| Elham S. | Mirfazeli |
