# Supplementary material for: Evolution of SARS-CoV-2 antibody repertoire after successive mRNA vaccinations under immunosuppressive treatment
Source: eBioMedicine. 2025 Feb 25;113:105620. doi: 10.1016/j.ebiom.2025.105620 (PMC11905820; doi:10.1016/j.ebiom.2025.105620)
Supplement: Supplementary file 2 — Supplementary Figures [file mmc2.pdf]

Supplementary material

"Evolution of SARS-CoV-2 antibody repertoire after successive mRNA vaccinations under immunosuppressive treatment", Keijser *et al.* 2024, eBioMedicine

Figure S1 2

Figure S2 3-4

Figure S3 5-6

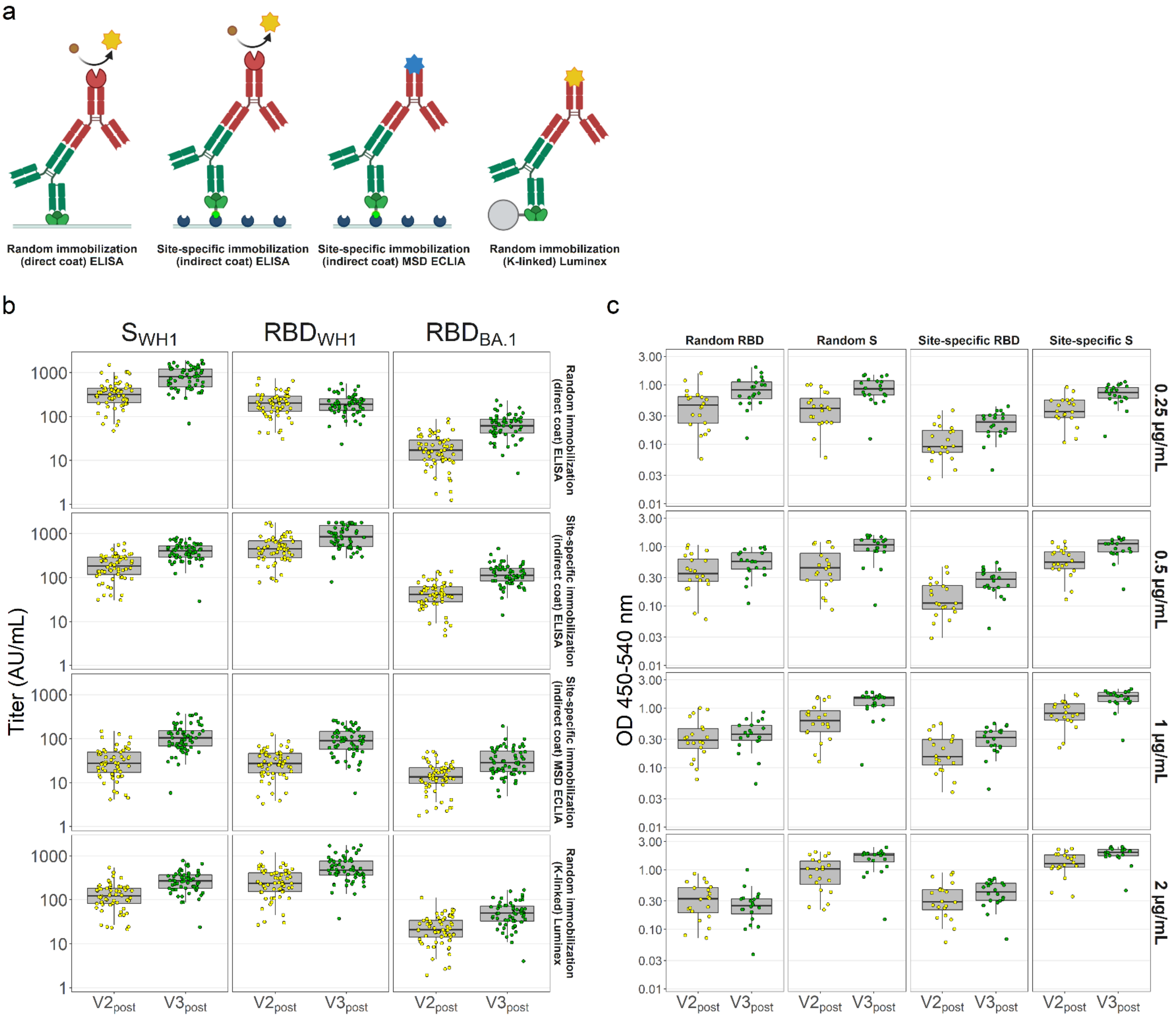

Figure S1: Impact of assay platforms and epitope density on serological antibody measurements

A subset of paired $V2_{post}$ – $V3_{post}$ samples from N = 60 healthy and disease control without infection before vaccination was retested using the main random immobilization ELISA format, a site-specific immobilization ELISA, another site-specific immobilization variant using an ECLIA readout, and a bead-based random immobilization variant. **a** Schematic overview of assay formats. **b** Box plots showing anti-WH1 S, anti-WH1 RBD, and anti-BA.1 RBD titers as measured in the assay formats described above. Titers were calculated in arbitrary units (AU) derived from pooled convalescent healthy donor plasma standards collected in early 2020 (WH1 standard) or early 2022 (BA.1 standard), which were set at 100 AU/mL. WH1, Wuhan-Hu-1; RBD, receptor-binding domain; S, spike (full protein). **c** Box plots showing anti-WH1 S and RBD raw signal (OD 450-540 nm, optical density at 450 nm minus at 540 nm) measured in the random and site-specific ELISA formats described above under different antigen coating concentrations, in an N = 20 subset of the samples shown in (**b**).

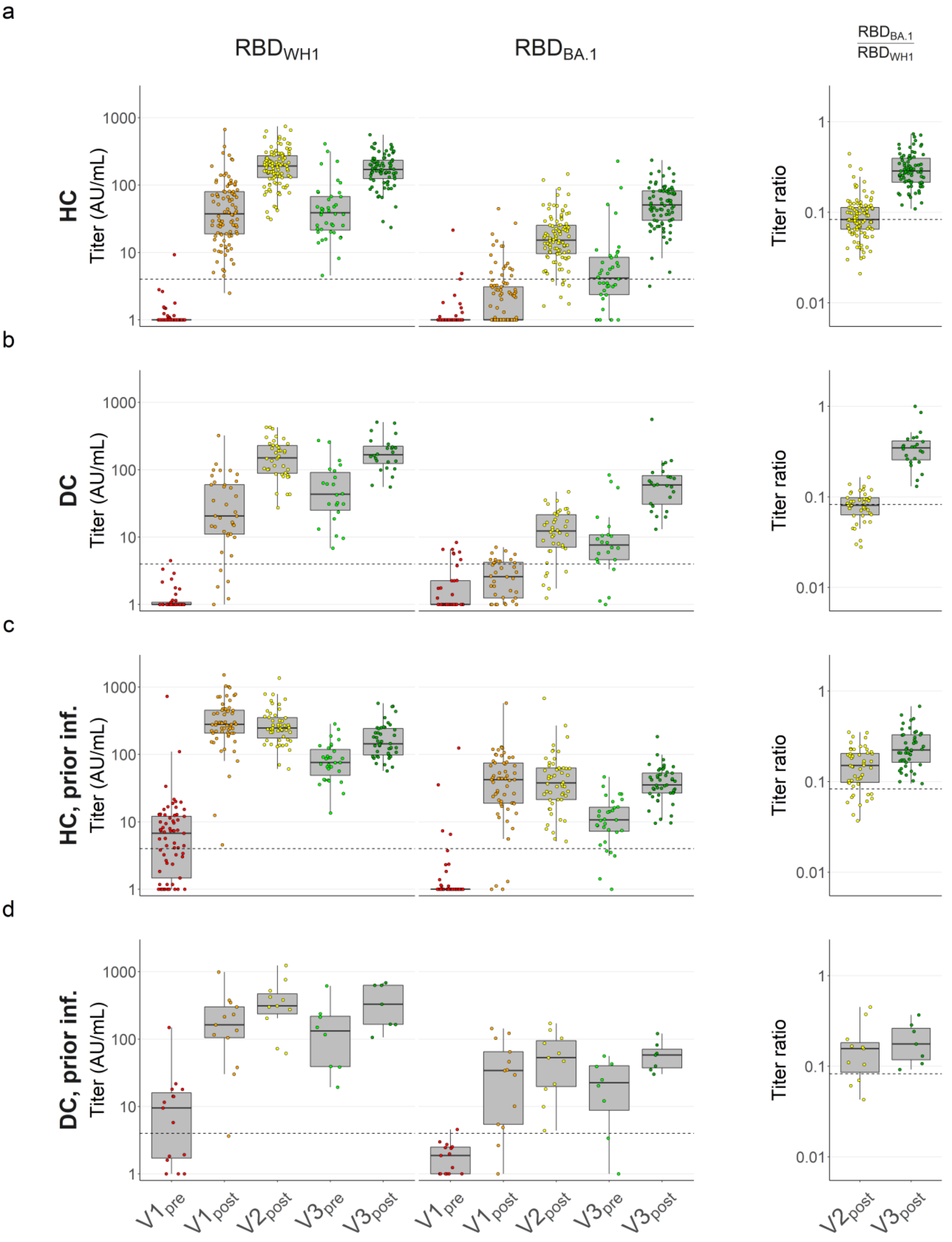

Figure S2: Serum IgG titers against SARS-CoV-2 WH1 and BA.1 RBD, WH1 S and BA.1 RBD in healthy and disease controls, separately, with and without prior infection.
Serum IgG concentrations were assessed by direct ELISA and titers were calculated in arbitrary units (AU) derived from pooled convalescent healthy donor plasma standards collected in early 2020 (WH1 standard) or early 2022 (BA.1 standard), which were set at 100 AU/mL. WH1, Wuhan-Hu-1; RBD, receptor-binding domain. **a-d** Box plots showing anti-WH1 RBD and anti-BA.1 RBD titers, and comparative ratios of healthy controls without infection

before vaccination (N = 118) and with prior infection (**b**, N = 73), disease controls without infection before vaccination (**c**, N = 53) and with prior infection (**d**, N = 19). Dashed lines in titer plots represent seropositivity cutoffs determined as the lowest integer AU value where >99% of pre-pandemic samples were considered negative. Dashed lines in ratio figures represent the median of naïve healthy controls (**a**) at $V2_{post}$ for comparison. In all box plots, central lines indicate the median, with hinges indicating 25th and 75th percentiles. Whiskers indicate the furthest data points up to 1·5 * IQR beyond hinges. V1, V2, V3; first, second and third vaccination.

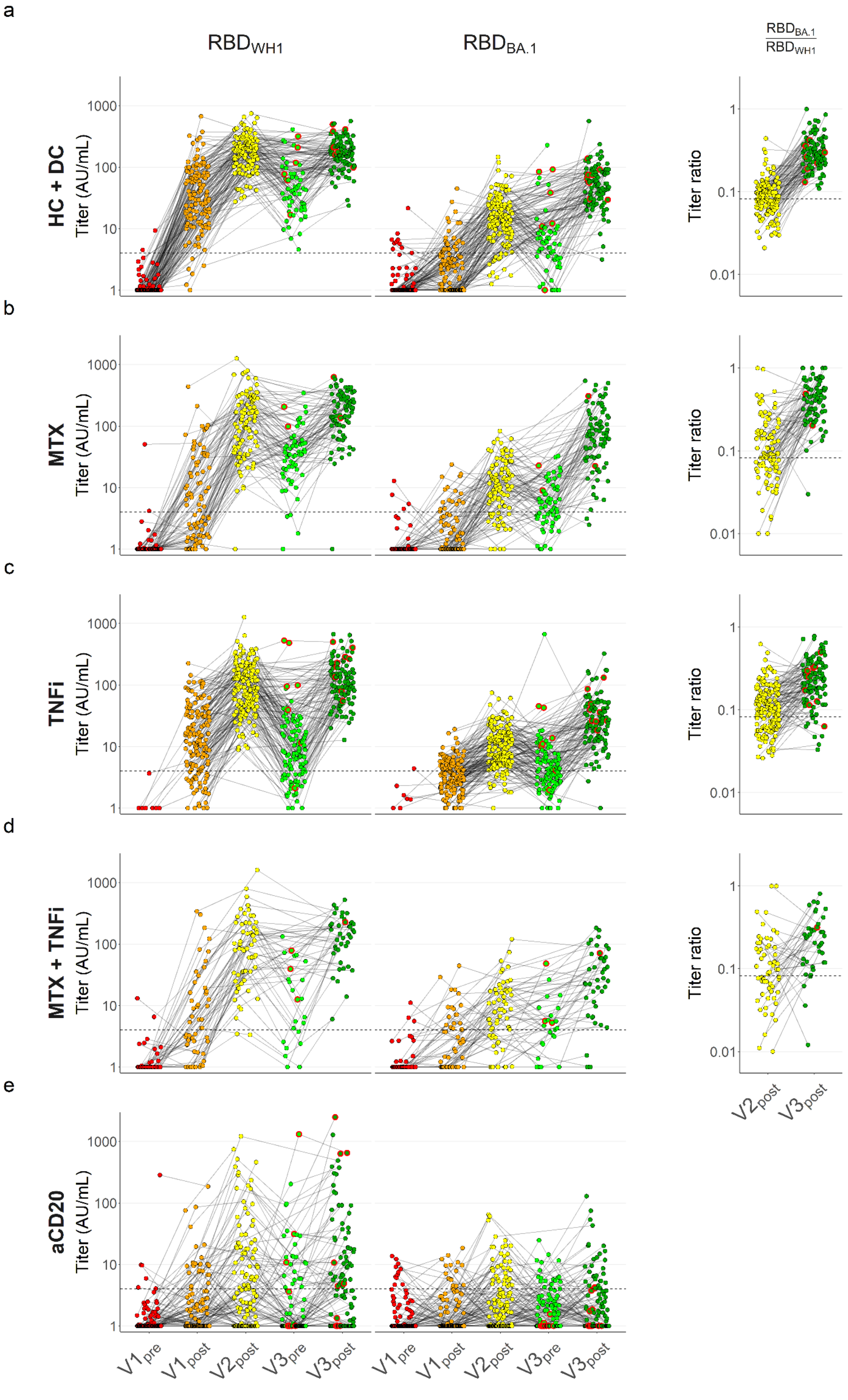
a
b
c
d
e
RBD_WH1
RBD_BA.1
RBD_BA.1 / RBD_WH1
HC + DC
MTX
TNFi
MTX + TNFi
aCD20
Titer (AU/mL)
Titer ratio
1000
100
10
1
0.1
0.01
V1pre
V1post
V2post
V3pre
V3post

Figure S3: Serum IgG titers against SARS-CoV-2 WH1 and BA.1 RBD, WH1 S and BA.1 RBD in healthy and disease controls, and patients under immunosuppressive treatment, with and without prior infection, highlighting breakthrough infections.
Serum IgG concentrations were assessed by direct ELISA and titers were calculated in arbitrary units (AU) derived from pooled convalescent healthy donor plasma standards collected in early 2020 (WH1 standard) or early 2022 (BA.1 standard), which were set at 100 AU/mL. WH1, Wuhan-Hu-1; RBD, receptor-binding domain. **a-e** Line plots showing anti-WH1 RBD and anti-BA.1 RBD IgG titers. Panel **a** corresponds to data shown in **fig. 3a**, **b** to **fig. 5a**, **c** to **fig. 5b**, **d** to **fig. 5c** and **e** to **fig. 5d**. Dashed lines in titer plots represent seropositivity cutoffs determined as the lowest integer AU value where >99% of pre-pandemic samples were considered negative. Bold red outlines indicate samples of participants who experienced a breakthrough infection after $V2_{post}$.
